# Ultrasound Detection of Portomesenteric Venous Gas Is an Early Sign of Bowel Ischaemia in Non-Traumatic Abdominal Pain: Old Dogs, New Tricks—Four Cases Report

**DOI:** 10.1155/2022/1734612

**Published:** 2022-08-23

**Authors:** Mirko Di Capua, Michela Tonani, Stefano Paglia

**Affiliations:** ^1^Emergency Department, “Maggiore” Hospital of Lodi, Largo Donatori del Sangue, 1, 26900 Lodi, Italy; ^2^Emergency Department, “Guglielmo da Saliceto” Hospital of Piacenza, Via Giuseppe Taverna, 49, 29121 Piacenza, Italy

## Abstract

Acute bowel ischemia is a severe disease often with a poor outcome. Early diagnosis can improve outcome, but atypical clinical manifestations and nonspecific laboratory and instrumental diagnostic findings may delay computed tomographic angiography (CTA). Portomesenteric venous gas (PVG), indirect sign of pneumatosis intestinalis, is considered a late finding with poor prognosis. We report four cases where PVG, easily identified through point-of-care ultrasonography (POCUS), was an early sign of bowel ischemia leading to a precocious diagnosis confirmed at CTA. In acute non-traumatic abdominal pain, an evidence of PVG could be an early ultrasonographic finding of bowel ischemia in the emergency department.

## 1. Introduction

Acute bowel ischemia is an uncommon cause of hospitalization, accounting for less than 1 out of 1000 admissions to hospital [1], but the overall mortality rate is approximately 70% where survivors of extensive bowel resection face lifelong disability. [2] Bowel ischemia is usually distinguished into acute mesenteric ischemia—meant as small bowel ischemia, venous mesenteric ischemia, non-occlusive mesenteric ischemia, and ischemic colitis. When hemodynamically stable ischemic colitis without gangrenous complication or perforation is suspected, colonoscopy is the gold standard and should be the first diagnostic test [3–4].

In the critical care setting, computed tomographic angiography (CTA) is considered the reference standard for diagnosis. [2]. Many authors have investigated the role of point-of-care ultrasonography (POCUS) in acute bowel ischemia [5, 6] and the European Federation of Societies for Ultrasound in Medicine and Biology (EFSUMB) recently published a position paper in intestinal emergencies. [7] Unfortunately, the intestinal ultrasonographic signs of ischemia are all nonspecific. Pneumatosis intestinalis is a well-recognized sign of bowel ischemia. It is, however, usually considered a late sign and difficult to evaluate with POCUS. Here, we describe four cases, admitted for acute non-traumatic abdominal pain, where POCUS detection of portomesenteric venous gas (PVG)—indirect sign of pneumatosis intestinalis—leads to a very early diagnosis of bowel ischemia.

## 2. Case 1

**Figure 1:**
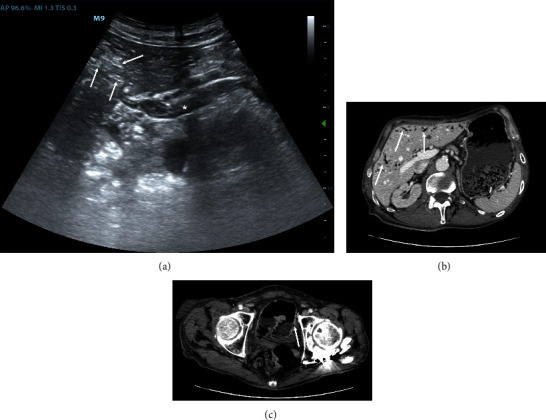
POCUS and CTA of case 1. (a) POCUS shows portal vein at hepatic hilum (asterisk) with multiple hyperechogenic spots into the lumen. White arrows indicate air in intrahepatic portal vessels with altered liver ultrasonographic structure. (b) A CTA scan confirms air into the liver (white arrows), while air is not easily identified into portal vein. (c) CTA confirms a little area of pneumatosis intestinalis (white arrow). POCUS stands for point-of-care ultrasonography; CTA stands for computed tomographic angiography.

Male, 56 years old. He was admitted to the emergency department (ED) with mild abdominal pain and hypotension. At arrival, heart rate (HR) was 77, blood pressure (BP) 88/65 mmHg, oxygen saturation (SAT) was 98% in room air, and body temperature was 36.3°C. Venous blood gas (VBG) analysis showed a normal acid-base balance with a mild lactate increase (2.25 mmol/L, normal value <2 mmol/L). POCUS showed an altered liver ultrasound pattern due to massive air infiltration in the portal system and portal vein showed a spontaneous echo-contrast with multiple hyperechoic spots flowing in the lumen suggestive of PVG (Figure 1(a)). We immediately performed a CTA that confirmed intestinal pneumatosis and diffuse air in the hepatic parenchyma (Figures 1(b) and 1(c)). The patient was admitted to the operating room and was found to have initial mesenteric ischemia due to several adhesions and a partial volvulus. Surgeons performed adhesions lysis and volvulus detorsion with success and regular post-operative progress. The patient was dismissed in 8 days.

## 3. Case 2

**Figure 2:**
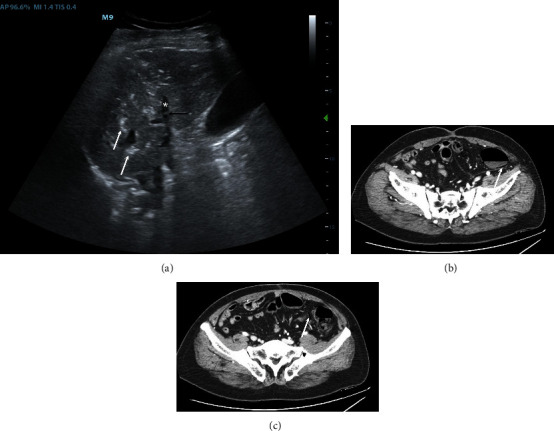
POCUS and CTA of case 2. (a) POCUS shows a right portal branch (asterisk) with some air bubbles into the lumen (black arrow). White arrows indicate some air in intrahepatic portal vessels. (b) CTA showing pneumatosis intestinalis (white arrow). (c) CTA shows the air flowing within the intestinal vessels (white arrow), as it is drained from the pneumatosis intestinalis, that will reach the portal vein. POCUS stands for point-of-care ultrasonography; CTA stands for computed tomographic angiography.

Male, 60 years old. He presented to the emergency department complaining of severe abdominal pain and vomiting. At evaluation, he presented a HR of 122 bpm, BP 112/70 mmHg, SAT 98% in room air, and body temperature of 36.3°C. Arterial blood gas (ABG) analysis showed a respiratory alkalosis and lactate within normal limits (0.95 mmol/L, normal value <2 mmol/L). Ultrasonography showed multiple hyperechogenic spots in the portal vein with altered liver ultrasonographic structure by diffuse air infiltration, compatible with PVG (Figure 2(a)). A CT angiography confirmed left colon pneumatosis and air in liver parenchyma (Figures 2(b) and 2(c)). The patient was admitted to surgery and laparotomy confirmed left colon ischemia complicated by peritonitis. Surgeons performed subtotal colectomy and an ileostomy. The patient was dismissed 30 days after surgical intervention.

## 4. Case 3

Female, 82 years old. She presented to the emergency department with severe acute abdominal pain, melena, and hypotension. At presentation, HR was 55 bpm, BP 80/40 mmHg, SAT 98% in ventimask with fraction of inspired oxygen of 0.4, and a body temperature of 36.2°C. A VBG showed a metabolic acidosis with hyperkalaemia (K 6.23 mmol/L), mild anaemia (haemoglobin 7.9 gr/dL), and normal lactates (1.35 mmol/L, normal value <2 mmol/L). Ultrasonography showed multiple hyperechogenic spots in the main portal vein, suggestive of PVG, and in intrahepatic portal vessels, we did not find free fluid. CT angiography confirmed intestinal pneumatosis and, moreover, showed diffuse mesenteric vascular impairment, particularly in the upper mesenteric artery. We decided not to proceed to the surgical intervention due to the high surgical risk. The patient died 16 hours after admission.

## 5. Case 4

Female, 88 years old. She was admitted to the emergency department complaining abdominal pain, haematochezia, and mild fever. At presentation, she had normal parameters (heart rate, blood pressure, and saturation) and mild fever (body temperature 37.4°C). ABG lactates were within normal limits. Ultrasound showed multiple hyperechogenic spots in intra- and extrahepatic portal vessels, suggestive of PVG. We performed an abdominal CT angiography which revealed ischemic colitis of the right colon and distal ileum. She was admitted to the operating room and a hemicolectomy with ileostomy was performed. The patient was dismissed after 7 days without complications.

## 6. Discussion

Acute bowel ischemia remains a severe disease with an often-poor prognosis. CTA is the reference standard diagnostic technique but it requires a high index of suspicion and so it is often performed at advanced stages of the disease. On the other hand, POCUS is widely available; it is usually performed immediately at bedside after physical examination. POCUS has demonstrated a high accuracy in several cardio-thoracic and abdominal emergencies. In some cases (e.g., pneumothorax), its specificity reaches 100%, while in other diseases, such as deep vein thrombosis, POCUS is considered the reference standard. As far as bowel ischemia is concerned, duplex ultrasonography has shown relatively high accuracy—both sensitivity and specificity of 85 to 90%—in diagnosing acute mesenteric ischemia, particularly when the upper mesenteric artery is involved by a thrombosis or an embolus [8, 9]. Unfortunately, it requires high degree of expertise in ultrasonography and its accuracy seems to be lower when distal vessels are responsible for ischemia or when there is a non-occlusive intestinal ischemia. On the other hand, ruling in or out bowel ischemia with only a basic POCUS evaluation is rarely considered safe as intestinal ultrasound has nonspecific patterns in bowel ischemia and inadequate accuracy [7].

Our small case series suggests that the association of acute non-traumatic abdominal pain—not explained by other emergencies—and ultrasound detection of PVG could be an indirect sign of bowel ischemia which deserves a rapid CTA confirmation. Even if the EFSUMB position paper cite the intestinal pneumatosis as a rare and late finding associated with poor prognosis, [7] our small series shows that even adhesions and a partial volvulus caused the development of severe early PVG. This indicates that pneumatosis intestinalis, and consequently PVG, does not need massive bowel ischemia or necrosis, but could rapidly develop also in segmental and initial ischemia. POCUS detection of PVG is quite easy and does not require high competence in ultrasonography. First, duplex ultrasound is not necessary. Moreover, basic competences are sufficient to confirm PVG as air is high visible on ultrasonography and an intercostal hepatic view allows evaluation of portal vessels and liver independent of meteorism or abdominal pain. Sonography may even have a higher sensitivity than CT in detection of gas in portal peripheral branches as Maher et al. have reported cases of PVG findings with POCUS, undetectable on CT performed immediately after an ultrasound evaluation. [10] Still, the evaluation of intestinal wall, to exclude or confirm the diagnosis of intestinal ischemia, needs higher competences; there is not a specific sign of intestinal ischemia and a negative ultrasonography does not exclude intestinal ischemia. The evidence of PVG at POCUS in a patient with acute onset of abdominal non-traumatic pain and the absence of other abdominal emergencies—gallbladder stones, cholecystitis, hydronephrosis, aortic aneurysm, acute urinary retention—increase the pre-test probability of bowel ischemia and deserves an early CTA. Well-conducted studies and wider sample size are necessary to demonstrate our hypothesis.

## Data Availability

The complete clinical records are available from the corresponding author upon request, for researchers who meet the criteria for access to confidential data.
